# Optimized Detection of *Plasmodium falciparum* Topoisomerase I Enzyme Activity in a Complex Biological Sample by the Use of Molecular Beacons

**DOI:** 10.3390/s16111916

**Published:** 2016-11-15

**Authors:** Asger Givskov, Emil L. Kristoffersen, Kamilla Vandsø, Yi-Ping Ho, Magnus Stougaard, Birgitta R. Knudsen

**Affiliations:** 1 Department of Molecular Biology and Genetics, Aarhus University, 8000 Aarhus C, Denmark; asgergivskov@gmail.com (A.G.); emillk@mbg.au.dk (E.L.K.); kwp93@live.dk (K.V.); 2 Division of Biomedical Engineering, Department of Electronic Engineering, The Chinese University of Hong Kong, Shatin, New Territories 999077, Hong Kong, China; ypho@ee.cuhk.edu.hk; 3 Department of Pathology, Aarhus University Hospital, 8000 Aarhus C, Denmark; magnus.stougaard@clin.au.dk

**Keywords:** real-time sensing, multiplexed detection, malaria, topoisomerase I, rolling circle amplification

## Abstract

The so-called Rolling Circle Amplification allows for amplification of circular DNA structures in a manner that can be detected in real-time using nucleotide-based molecular beacons that unfold upon recognition of the DNA product, which is being produced during the amplification process. The unfolding of the molecular beacons results in a fluorescence increase as the Rolling Circle Amplification proceeds. This can be measured in a fluorometer. In the current study, we have investigated the possibility of using two different molecular beacons to detect two distinct Rolling Circle Amplification reactions proceeding simultaneously and in the same reaction tube by measurement of fluorescence over time. We demonstrate the application of this fluorometric readout method, for automated and specific detection of the activity of the type IB topoisomerase from the malaria parasite *Plasmodium falciparum* in the presence of human cell extract containing the related topoisomerase I from humans. The obtained results point towards a future use of the presented assay setup for malaria diagnostics or drug screening purposes. In longer terms the method may be applied more broadly for real-time sensing of various Rolling Circle Amplification reactions.

## 1. Introduction

DNA-based sensors and detection systems have in recent years been increasingly used in relation to detection of multiple DNA-modifying enzymes such as topoisomerases, nucleases and DNA repair enzymes [1,2,3,4,5,6,7,8,9]. In many cases, specially designed DNA oligonucleotides have been used to detect the enzymatic reaction by allowing for specific amplification of the DNA oligonucleotides, only after the correct enzymatic reaction has been performed [5,6,7,8,9,10]. These systems enable ultra-sensitive (down to the single-enzymatic-event-level) detection of enzymatic reactions. Various strategies for amplification of DNA structures have been established, including Polymerase Chain Reaction (PCR) [11,12], exonuclease III assisted signal amplification [13], strand displacement amplification [14], catalytic hairpin assembly [15] and Rolling Circle Amplification (RCA) [9,16,17,18,19,20]. Among the mentioned techniques, RCA has been widely utilized in DNA oligonucleotide-based enzyme activity detection [5,7,8,9,21].

RCA is defined by the isothermal polymerase directed amplification of a circular DNA template, where the newly synthesized DNA is displaced in front of the polymerase as it moves around the template [22,23,24,25]. Using the highly processive Phi29 DNA polymerase to catalyze RCA, it is possible to produce a long multiple-repeats-product, termed the Rolling Circle Product (RCP) [16,23]. Two main strategies for measuring the RCA reaction have been described: real-time detection that occurs during the amplification, or end-point measurement of the number of generated RCPs [5,9,25,26,27,28,29,30,31]. As an example of the later, RCPs have been immobilized on microscopic slides and labeled by the hybridization with sequence-specific fluorescent probes. Using this approach, termed the microscopic readout in the following, each RCP can be detected as a fluorescent spot under a fluorescence microscope [7,8,22]. The microscopic readout is ultra-sensitive but very labor intensive. The method demands specialized equipment and is difficult to automate. As alternative methods, RCA can be measured in real-time using DNA intercalating dyes [32] or molecular beacons [33,34,35]. Such approaches are much quicker than the microscopic approach and can easily be standardized allowing for easy analyses of multiple samples.

Previously, we described a set of DNA oligonucleotide substrates for enzyme detection for various diagnostic purposes using a technique called Rolling circle Enhanced Enzyme Activity Detection (REEAD) [8,9]. We demonstrated that the utilized DNA oligonucleotide substrates (in the present study termed the dumbbell substrates) were specifically circularized by their target enzymes e.g., DNA topoisomerase I from human (hTopI) or from the malaria parasite *P. falciparum* (pfTopI). This allowed for RCA of the generated circles and detection using the fluorescence microscopic readout method, as described above. Topoisomerase I is an ubiquitous and essential enzyme that is taking part in maintaining the genomic topology, by relaxing helical tension through a swivel based mechanism including transient single stranded nicking of the DNA double helix [36]. Moreover, the human enzyme is the cellular target of important anti-cancer drugs from the camptothecin family and studies have demonstrated a direct correlation between hTopI activity and cellular drug response [37,38,39]. In line with this, using the REEAD approach, it has been possible to predict the camptothecin response of even very rare cell populations [40]. Measurement of pfTopI by REEAD, on the other hand, was successfully employed for diagnosis of malaria using a single drop of blood from infected individuals [9]. In line with the high sensitivity of REEAD, the protocol for malaria diagnosis was by far superior to other known methods with regard to detection limit and may, hence, hold great promise as a new malaria diagnostic tool provided that readout can be optimized for low-resource-settings, where malaria cases are predominant.

**Figure 1:**
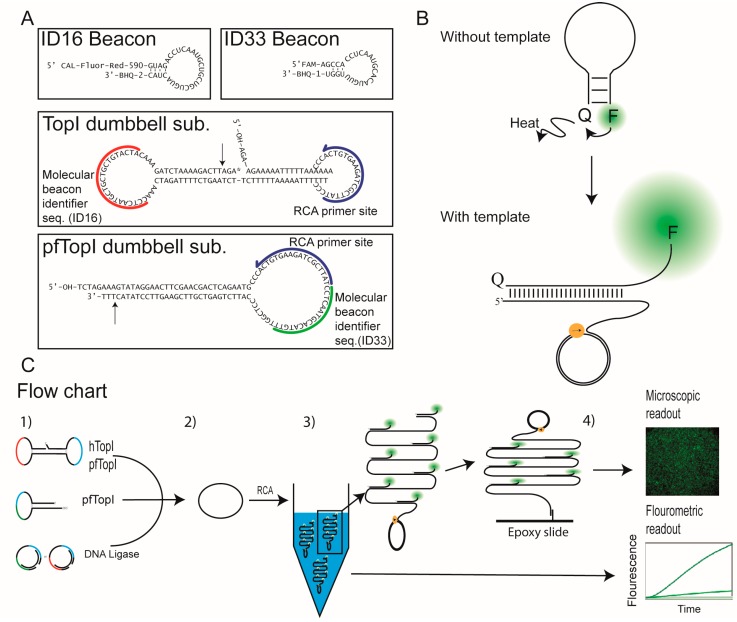
(**A**) Shows the secondary structure and sequence of molecular beacons and dumbbell substrates. Blue line represents the primer, which are used for initiation of the RCA reaction. The 3′-OH end of the primer is illustrated by an arrow head. Red and green lines represent the position of the molecular beacon indentifier sequence specific for the ID16 and the ID33 beacon, receptively. The indentifier sequences are identical to the annealing parts of molecular beacons. Therefore the RCPs generated by RCA of circularized dumbbell substrates will contain sequences complementary to the respective matching molecular beacons; (**B**) Schematic illustrations of the secondary structures of the molecular beacons in the absence or presence of a matching template sequence. In the absence of matching template the molecular beacons form a hairpin structure bringing the fluorophore and quencher into close proximity. This results in quenching of the fluorescence (upper panel). In the presence of matching template, the molecular beacons unfold to hybridize with the template, resulting in separation of the fluorophore and quencher and an increase in fluorescence (lower panel). (**C**) Flow chart showing the assay setup. (**1**) Schematic depiction of the TopI dumbbell substrate that can be circularized by hTopI and pfTopI, the pfTopI dumbbell substrate that can be circularized by pfTopI, and the control circles (c.c.) that can be circularized by a DNA ligase; (**2**) DNA circles are generated upon incubation with the appropriate enzymes; (**3**) Circles are amplified by RCA leading to long tandem repeat RCPs. The RCPs are visualized upon hybridization with the molecular beacons; (**4**) The assay has two readout formats: a fluorometric readout and a fluorescence microscope readout. The fluorometric readout measures development of fluorescence as the RCPs increase in size over time. The microscopic readout quantifies the number of RCPs hybridized to the surface of an epoxy slide functionalized with a capture oligonucleotide.

In order to setup an optimized readout approach for the REEAD assays, as an alternative to the current microscopic readout format, we designed a fluorometric readout format based on molecular beacons for multiplexed detection of the RCA of the circularized dumbbell substrates (see schematic outline of the assay setup in Figure 1). We show that the molecular beacons can be used for multiplexed and specific detection of two RCA reactions running in the same reaction tube. Furthermore, we demonstrate that the beacons can be applied for REEAD based specific detection of pfTopI in a background of complex biological samples, i.e., human cell extracts.

## 2. Materials and Methods

### 2.1. Oligonucleotides, Substrates and Molecular Beacons

All oligonucleotides were prepared by DNA Technology (Aarhus, Denmark).

#### 2.1.1. Oligonucleotides for Preparation of c.c.

Oligonucleotide 1.1 (ID16): 5′-GGA AGA GAT GGC GAC ATC ATC GAT CGG TCG GCA CCG GAT CCC TGC AGG CTG AGG ATA AGC GAT CTT CAC AGT TAC GAA CTG ACC TCA ATG CTG CTG CTG TAC TAC AGC TGA TCC TGA TGG-3′.

Oligonucleotide 1.2 (ID33): 5′-GGA AGA GAT GGC GAC ATC ATC GAT CGG TCG GCA CCG GAT CCC TGC AGG CTG AGG ATA AGC GAT CTT CAC AGT TAC GAA CTG ACC TCA ATG CAC ATG TTT GGC TCC AGC TGA TCC TGA TGG-3′.

Oligonucleotide 2 (ligator oligonucleotide): 5′-GTC GCC ATC TCT TCC CCA TCA GGA TCA GCT-3′.

#### 2.1.2. Dumbbell Substrates

TopI substrate: 5′-AGA AAA ATT TTT AAA AAA CCC ACT GTG AAG ATC GCT TAT CCC TTT TTT AAA AAT TTT TCT AAG TCT TTT AGA TCA AAC CTC AAT GCT GCT GCT GTA CTA CAA AGA TCT AAA AGA CTT AGA-3′.

pfTopI substrate: 5′-TCT AGA AAG TAT AGG AAC TTC GAA CGA CTC AGA ATG CCC ACT GTG AAG ATC GCT TAT CCT CAA TGC ACA TGT TTG GCT CCC ATT CTG AGT CGT TCG AAG TTC CTA TAC TTT-3′.

RCA primer for dumbbell substrates: 5′-ATA AGC GAT CTT CAC AGT-3′.

#### 2.1.3. Molecular Beacons

ID16 beacon: (2′OMe-RNA) 5′-CAL-Fluor-Red-590 (TAMRA analog)-GUA GAC CUC AAU GCU GCU GCU GUA CUA C-BHQ-2 (Black Hole Quencher-2)-3′.

ID33 beacon: (2′OMe-RNA) 5′-FAM-AGC CAC CUC AAU GCA CAU GUU UGG U- BHQ1 (Black Hole Quencher-1)-3′.

### 2.2. Preparation of Control Circles

The oligonucleotides for preparation of the control circles were phosphorylated by T4 polynucleotide kinase in 1 × T4 DNA ligase buffer containing 50 mM Tris-HCl (pH 7.5), 10 mM MgCl_2_, 1 mM adenosine triphosphate (ATP), 10 mM dithiothritol (DTT) and 1 mM freshly prepared ATP for 30 min at 37 °C. Subsequently the samples were heat inactivation for 20 min 65 °C. Then, the control circles were ligated by mixing oligonucleotides 1.1 (ID16) with oligonucletiode 2 (ligator) and oligonucleotide 1.2 (ID33) with oligonucleotide 2 (ligator) in a 1:10 ratio, respectively, before 200 units of T4 DNA ligase was added and incubated performed for 30 min at 37 °C. Before use, the control circles were diluted to the desired concentration in 1 × TE (10 mM Tris-HCl (pH 7.5), 1 mM ethylenediaminetetraacetic acid (EDTA)).

### 2.3. REEAD Experiments

REEAD experiments were performed by mixing 95 nM of the pfTopI dumbbell substrate and 5 nM of the TopI dumbbell substrate in 1 × TE + E buffer (10 mM Tris-HCl (pH 7.5) and 5 mM EDTA) followed by addition of pfTopI, hTopI or cell extract as specified in the bulk text or in the figures (note that the TopI dumbbell substrate is a much better substrate for the topoisomerases compared to the pfTopI dumbbell substrate (data not shown), therefore, in order to boost the pfTopI dumbbell substrate reaction, the 19:1 ratio of pfTopI and TopI dumbbell substrate, respectively, was used as noted above). NaCl concentrations were as noted in the text. After addition of the enzyme and/or cell extract, samples were incubated at 37 °C for 1 h if nothing else has been noted. The enzymatic reactions lead to circularization of the dumbbell substrates. The reactions were terminated by heat inactivation (95 °C for 20 min) and samples were hereafter stored on ice until further analysis. Finally, addition of a large excess of primer (200 nM) prepared the dumbbell substrates for RCA.

### 2.4. RCA and Fluorometric Analysis

7.5 µL of the noted concentrations of c.c. or reacted dumbbell substrates were moved to quantitative PCR (qPCR) tubes and the buffer adjusted to 1 × RCA buffer (33 mM Tris-acetate (pH 7.9), 10 mM Mg-actate, 66 mM K-acetate, 1% (*v*/*v*) Tween 20, and 10 mM DTT). Then 15 nM Phi29 Polymerase (ThermoFisher Scientific, Waltham, MA, USA), 1 mM dNTP (Roche , Basel, Switerland), 0.1 µg/µL BSA (Sigma, Saint Louis, MO, USA), and 1 µM of each of the molecular beacons were added to a final volume of 10 µL. The samples were placed in the qPCR machine (Mx3000P, Agilent Technologies, Inc., Santa Clara, CA, USA) and incubated at 30 °C for up to 18 h. Fluorescence emission was measured every 1 min using the FAM (U-FBNA FL filter cube (Excitation: BP470-495, DM505, Emission: BP510-550) and TAMRA (U-FGNA FL filter cube (Excitation: BP540-550, DM570, Emission: BP575-625). The TAMRA filter was used for CAL-Fluor-Red-590. Data from the run was processed in Microsoft Excel as described below.

### 2.5. Microscopic Analysis

The Epoxy slides (Arrayit, Sunnyvale, CA, USA) were functionalized by binding of the amino capture oligonucleotide according to the manufacturer’s protocol. The RCPs obtained from the amplification in the qPCR machine were hybridized to the coupled primer and the microscopic pictures were acquired as previously reported [41]. Briefly, the slides were mounted with 2.5 µL Vectashield (Vector Laboratories, Burlingame, CA, USA) and a cover glass. The pictures were acquired using a fluorescence microscope (Olympus IX73, Olympus Europa Holding GMBH, Hamburg, Germany) equipped with a 63× objective and a camera. A total of 12 pictures per slide were acquired and analyzed using (free) ImageJ software to count the fluorescent spots.

### 2.6. Expression and Purification Enzymes

The yeast *Saccharomyces cerevisiae* Top1 null strain RS190 was a kind gift from R. Sternglanz (State University of New York, New York, NY, USA). Plasmid for expression of recombinant full-length hTopI and pfTopI in yeast and purification of the expressed enzymes were described previously [42,43].

### 2.7. Preparation of Cell Extracts

HeLa cells used to make extracts were grown under appropriate conditions, harvested and stored as cell pellets at −80 °C. When extract was needed, cell pellets were thawed on ice followed by incubation on ice for 10 min in lysis buffer (0.1% Igepal (CA-630), 10 mM Tris-HCl pH 8, 20 mM MgCl_2_ 15 mM NaCl) (1 mL/2 × 10^6^ cells). The samples were centrifuged at 2200 rpm for 5 min. The supernatant was discarded and extraction buffer (0.5 M NaCl, 20 mM HEPES (4-(2-hydroxyethyl)-1-piperazineethanesulfonic acid) pH 8, 20% *v*/*v* glycerol) was added to the pellet followed by rotation of the sample for 1 h at 4 °C. Finally, the samples were centrifuged at 11.000 rpm for 10 min and the supernatants collected. Freshly prepared PMSF (Phenylmethanesulfonyl Fluoride) dissolved in isopropanol was added every 15 min (1/1000 of the total volume each time). Nuclear extracts was always freshly prepared and stored on ice maximally 1h before use. In the presented experiments 1 million cells were used to prepare 20 µL extract, resulting in 50.000 cells/µL.

### 2.8. Data Processing

**Figure 2:**
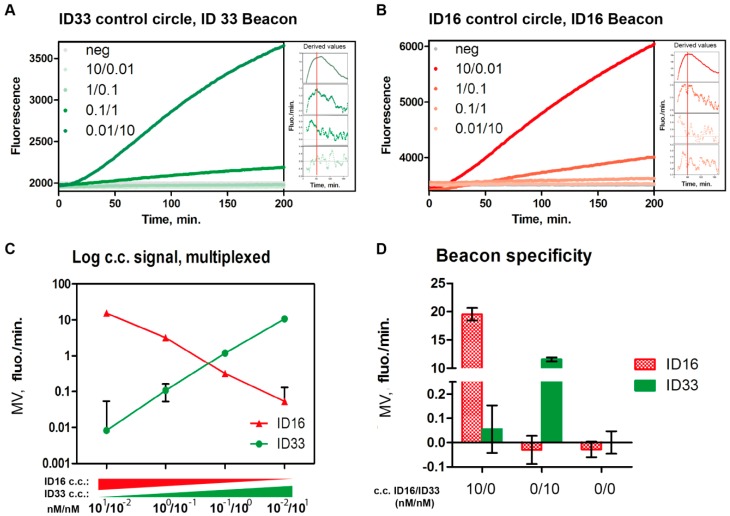

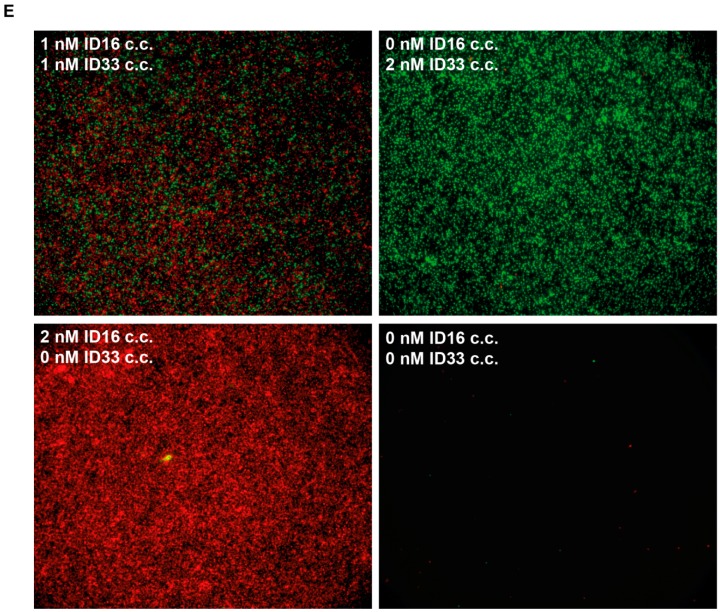
(**A**) Shows the mean of primary results obtained when measuring FAM fluorescence as a function of time in RCA reactions performed on mixtures containing 10 and 0.01 nM, 1 and 0.1 nM, 0.1 and 1 nM, or 0.01 and 10 nM of the ID16 and ID33 containing c.c., respectively, in the presence of molar surplus of the ID16 and the ID33 molecular beacons; (**B**) Same as A except that CAL-Flour-Red-590 fluorescence was measured; (**C**) Graphical depiction showing the mean of the MV of RCA of each of the c.c. calculated from the experiments shown in (**A**,**B**). Error bars represent standard deviation (SD) calculated from three individual experiments. MV for RCA of either c.c. was calculated after 1 h. Green circles represent FAM fluorescence detected per min (arising from ID33 beacon unfolding), and red triangles show CAL-Flour-Red-590 fluorescence per min (arising from ID16 beacon unfolding); (**D**) Bar chart showing the mean MV of triplicate experiments where only one of the two c.c.s or none of them was added to the RCA mixture before readout. Both FAM (green bars) and CAL-Flour-Red-590 (red bars) fluorescence were measured for each sample and the results plotted in the figure. Note that the y-axis is broken and shows the interval of −0.1 to 0.25 and of 10 to 25 (fluo/min). Error bars represent SD of triplicate experiments. MV for RCA of either c.c. was calculated after 1 h; (**E**) Representative fluorescence microscopic images obtained when analyzing the samples containing the indicated ratios of the ID33 or ID16 containing c.c. using the microscopic readout. Green spots represent FAM fluorescence arising from binding of the ID33 beacon to RCPs resulting from RCA of the ID33 containing c.c. Red spots represent CAL-Fluor-Red-590 fluorescence arising from binding of the ID16 beacon to RCPs resulting from RCA of the ID16 c.c.

The primary data (in the form of arbitrary fluorescence units) was extracted from the qPCR machine, loaded into Excel (Microsoft, Redmond, WA, USA) and plotted as a function of time to generate the primary data plots. Derived values from the primary data were achieved by calculating the slope of linear least square fits (using 29 data points) for all time points in steps of one. The derived values were plotted as a function of the mean of the time points used to make the linear fit. This gave rise to the derived plots. The derived plots were used to identify T_MV_ by manually identifying the peak value in the plot. In order to achieve robust calculations of MV, a new least square fit (using a total of 59 data points) were made to the primary data flanking the identified T_MV_. The slope of the robust least square fit was used as MV. For samples where T_MV_ could not be identified in the derived plots (such as the case for derived plots of the low c.c. concentration experiments shown in Figure 2A,B), the MV was calculated using the same time as the samples with higher concentrations. Figures were prepared by GraphPad Prism. Statistical test was performed by GraphPad Prism (Two-tailed unpaired *t* test).

## 3. Results

### 3.1. Design of the Molecular Beacons and REEAD Setup

As described above, molecular beacons can be used to detect the RCA reaction in real-time by measuring increase in fluorescence over time using a fluorometer or equivalent e.g., a qPCR machine, which was used in the current study. The detection is quantitative and the increase in fluorescence over time has previously been demonstrated to be proportional to the amount of circularized template (resulting from the enzymatic reaction) [34]. The molecular beacons used here were made of 2′-OMe RNA oligonucleotides (to protect against nuclease digestion in crude biological samples and the exonuclease activity of the Phi29 polymerase) and had a fluorophore attached to the 5′-end and a quencher attached to the 3′-end (see Figure 1A). The molecular beacons were termed ID16 beacon (having a TAMRA-like CAL-Fluor-Red-590 fluorophore) and ID33 beacon (with a FAM fluorophore), respectively. Figure 1A shows the nucleotide sequences and the proposed secondary structures of the TopI and pfTopI specific dumbbell substrates used in the REEAD setup. The dumbbell substrates contained a preferred cleavage site for either pfTopI (pfTopI dumbbell substrate) or both pfTopI and hTopI (TopI dumbbell substrate) as well as a primer annealing site (primer indicated in blue with the direction (i.e., the position of the 3′-OH end) shown by the arrow) and a molecular beacon identifier sequence (indicated in green or red). Upon reaction with hTopI and/or pfTopI the dumbbell substrates were circularized as described in [8,9] and could thereby template RCA mediated by the highly processive Phi29 polymerase to generate long-tandem-repeat RCPs containing a sequence matching one of the utilized molecular beacons. In the absence of correct template the sequence of the molecular beacon promoted formation of a short 3–4 bp stem-loop structure in solution. This placed the fluorophore and quencher in very close spatial proximity (see Figure 1B, without template) and the fluorophore emission was quenched. In the presence of a correct template (the correct RCP), the beacon would be able to unfold by hybridization to the template. This resulted in separation of the fluorophore and quencher and gave rise to an increase in fluorescence (see Figure 1B, with template). Beacon designs were based on the principle previously described by Nilsson et al. [34] in such a way that upon template binding the fluorophore would be displaced as far away from the quencher as possible at the terminal of a single stranded “flap” region. This would ensure maximum fluorescence from the fluorophore by placing it away from the DNA duplex as well as from the quencher (see Figure 1B).

Note, that if the dumbbell substrate was not circularized by its target enzyme(s), extension of the primer by the Phi29 polymerase would be terminated by the strand-interruption in the substrate and consequently an annealing site for the molecular beacon would not be generated (see Figure 1A). Hence, no signal would be generated in the absence of target enzyme. Indeed the specificity of the dumbbell substrates and the lack of Phi29 polymerase mediated RCA in the absence of the designated target enzyme was confirmed previously [7,9].

### 3.2. The Use of Molecular Beacons for Readout Allows for Multiplexed Detection of Two RCA Reactions Performed in the Same Test Tube

The possibility of detecting two different RCA reactions in the same test tube by using the ID16 and the ID33 molecular beacons for readout (as schematically depicted in Figure 1C) was investigated by using a known concentration of preformed DNA control circles (c.c.) to template RCA. The c.c.s were designed with an ID16 or an ID33 molecular beacon identifier sequence. To ensure a linear correlation between fluorescent signals arising from molecular beacon hybridization to the RCPs and the utilized c.c. concentrations, the c.c.s were mixed with molar excess of Phi29 polymerase as well as high excess of molecular beacons in all experiments described below.

To address the possibilities of quantitative multiplexed detection of two RCA reactions even when the templates for RCA differ markedly in concentration, the ID16 and ID33 containing c.c.s were cross-titrated from 10 to 0.01 nM of each c.c. in steps of 10 times dilution, before RCA and detection were performed using the molecular beacons as described above (and outlined in Figure 1C).

Figure 2A,B show primary results from such titration experiments, in which the lowest amount of the ID16 containing c.c. was mixed with the highest amount of the ID33 containing c.c. and vice versa. For all samples, the fluorometer was set up to measure both CAL-Fluor-Red-590-fluorescence (Figure 2A,B, red lines) arising from unfolding of the ID16 beacon and FAM-fluorescence (Figure 2A,B, green lines) resulting from unfolding of the ID33 beacon. The inserts in Figure 2A,B, show the derived values from the datasets calculated by making linear fits to the primary data curve in successive time intervals of 0–30 min, 1–31 min and so on, and subsequently plotting these as a function of the mean time of the intervals (these inserts are shown in increased size in Figure S1).

Together, these figures illustrate the progression of RCA over time as measured in terms of increase in fluorescence emission resulting from unfolding of the molecular beacons. From the plots of the derived values the optimal time for data analysis (indicated by the peak) could be determined. From the peak (see Figure S1), the Maximal reaction Velocity (MV) and the time at which MV was reached (T_MV_) were obtained as described in the Experimental section. In praxis we used the derived plots to manually identify the T_MV_. MV was then calculated by calculating the slope of a more robust linear fit (consisting of 60 measure points) of the primary data around T_MV_. Note all results in the present study were determined as MV of the RCA reaction since this gave a very robust quantitative measure of the relative concentration of a given circularized substrate present in the solution under investigation. As indicated by Figure 2A,B, end-point measurements could also be used for quantitative measurements of c.c. concentrations but may be less precise when measuring subtle variations in circle substrate concentrations. The MVs obtained from the multiplexing experiments in which RCA of the two different c.c. were analyzed are plotted in Figure 2C. From the graph it is evident that the plotted MVs (representing FAM fluorescence per min or CAL-Fluor-Red-590 fluorescence per min) correlate proportionally with the amount of ID33 and ID16 c.c.s added to the reaction mixture, respectively. This indicates that the RCA of the two c.c.s can be measured simultaneously using the molecular beacons and that the signals achieved correlate to the concentration of c.c. template in the sample. The plotted MV values were calculated at around one hour (T_MV_: ~1 h).

The beacon specificity was further investigated by performing experiments where only one or the other c.c. was added before RCA was performed in the presence of both molecular beacons. Results from these experiments are plotted in the bar chart (Figure 2D) showing the mean MV calculated for FAM fluorescence (arising from unfolding of the ID33 beacon) and for CAL-Fluor-Red-590 fluorescence (arising from unfolding of the ID16 beacon). Paired columns (marked 10/0, 0/10 or 0/0) represent data obtained by measuring each of the fluorophore (CAL-Fluor-Red-590 or FAM) emissions generated in a single reaction tube in three individual repetitions. From the plot, it is evident that only the expected signal was detected above the background. This demonstrated a high degree of specificity, and indicated that the system could indeed be used for multiplexed detection of RCA of two different DNA circles. Note that the signal coming from RCA of 10 nM of the ID16 containing c.c. was slightly lower in the presence of 0.1 nM of the ID33 containing c.c. compared to when no other c.c. was added. Hence, MV was 16.4 fluo/min for 10 nM of the ID16 containing c.c. when analyzed in the presence of 0.1 nM of the ID33 containing c.c. (Figure 2C and Figure S1B), and 19.6 fluo/min when analyzed alone (Figure 2D, first column). The reason for this difference is not clear since all experiments were performed in the presence of large excess of polymerase and nucleotides. However, the difference is rather modest and could reflect random variations.

As mentioned above (and shown in Figure 1C) samples that are first analyzed in the fluorometer can also be analyzed by a fluorescence microscope [35] simply by hybridizing the beacon-bound RCPs to microscopic slides functionalized with appropriate oligonucleotides. Since readout by the microscope is considerably more sensitive than in a fluorescence reader this approach can potentially be used in cases where such high sensitivity is necessary (see also [35]). In previous investigations the feasibility of the approach was only investigated for one molecular beacon and one RCP at a time. To test if a tandem readout (fluorometric readout followed by microscopic readout) was also possible when detecting two different RCPs with two different molecular beacons simultaneously, a multiplexing experiment was performed to investigate the specificity of the molecular beacons in the ultra-sensitive microscopic readout format. In the experimental setup different amounts of the two different c.c.s each containing either the ID16- or the ID33 specific molecular beacon identifier sequence were mixed and amplified by RCA in the presence of both the ID16- and the ID33 beacons. After RCA, the reaction mixtures were moved to primer coated microscopic slides and analyzed by the fluorescence microscope. Representative images obtained by this analyses are shown in Figure 2E. From the images it is evident that both red (CAL-Fluor-Red-590 fluorescence) and green (FAM fluorescence) spots representing RCPs recognized by either the ID16- or the ID33 beacon were detected from the reactions with 1 nM of each c.c. (upper right image). However, consistent with specific detection, only red (coming from unfolding of the ID16 beacon) or green (resulting from unfolding of the ID33 beacon) spots were detected in the reaction mixtures containing selectively 2 nM of either the ID16 or the ID33 containing c.c. Finally, only trace number of “background” spots was observed in the samples without any c.c. added. Hence, the results presented in Figure 2E strongly support the possibilities of tandem multiplexed analyses of the same sample using the microscopic readout followed by a fluorescence reader.

### 3.3. Molecular Beacon Based Real-Time Detection of RCA Performed on TopI Generated DNA Circles

**Figure 3:**
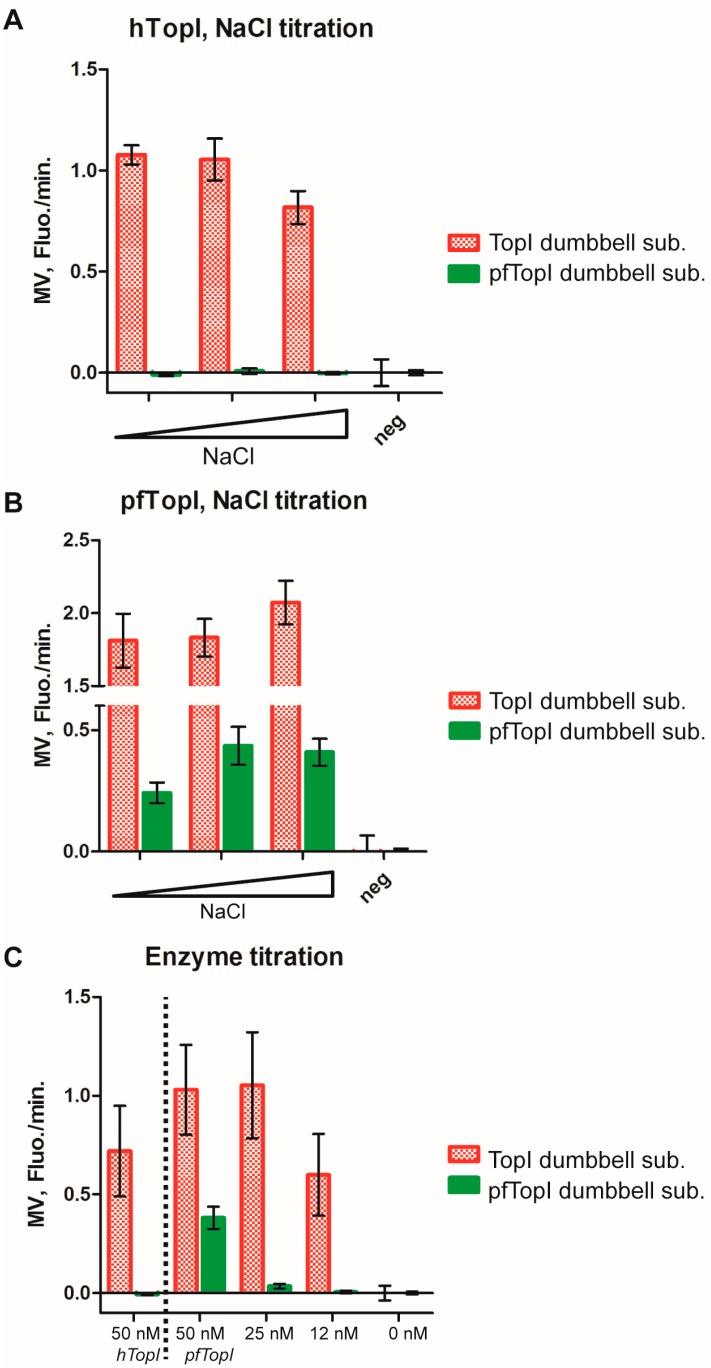
(**A**) Bar chart showing mean MV from six experiments where dumbbell substrate mixtures containing 95 nM pfTopI dumbbell substrate and 5 nM TopI dumbbell substrate (termed dumbbell substrate mixtures) were incubated with 50 nM hTopI at different NaCl concentrations (100, 150 and 200 mM) and analyzed by RCA and fluorometric readout. The negative control (neg) represents a sample without added enzyme and with 200 mM NaCl. Error bars represent Standard Error of the Mean (SEM) calculated from six experiments; (**B**) Same as (**A**) except that the dumbbell substrate mixture were incubated with 50 nM pfTopI instead of hTopI; (**C**) Bar chart showing mean MV from six experiments where dumbbell substrate mixtures were incubated with the noted type and amount of enzyme and analyzed as described in (**A**). Error bars represent SEM calculated from six experiments. For all charts red bars represent CAL-Fluor-Red-590 fluorescence arising from ID16 beacon binding to RCPs generated from circularized TopI dumbbell substrate. Green bars show FAM fluorescence resulting from ID33 beacon binding to RCPs generated from the circularized pfTopI dumbbell substrate.

As previously described, the activities of pfTopI or hTopI have been specifically detected in complex biological samples for e.g., diagnostic purposes using the REEAD technique in combination with the microscopic readout method [7,8] This was based on selective circularization of special designed dumbbell substrates (Figure 1A) by the target enzymes followed by RCA and visualization. More detailed, the dumbbell substrate termed TopI substrate was used to detect both hTopI and pfTopI [8], and is considered to be a general substrate for the type IB family of topoisomerases. In contrast, the pfTopI dumbbell substrate appears highly specific for pfTopI at least in human samples such as blood, saliva or human cell extracts [9]. For the present study the TopI dumbbell substrate was designed with an ID16 molecular beacon identifier sequence. Hence, RCPs resulting from RCA of the circularized TopI dumbbell substrate were recognized by the CAL-Fluor-Red-590 labeled ID16 beacon. The pfTopI dumbbell substrate contained an ID33 molecular beacon identifier sequence and, consequently, RCPs generated by RCA of the circularized pfTopI dumbbell substrate were recognized by the FAM labeled ID33 beacon. It was previously demonstrated that the TopI dumbbell substrate is a much better substrate for pfTopI than the pfTopI dumbbell substrate, which on the other hand is specific for pfTopI and unable to react with hTopI [8,9]. To counteract the different efficiencies by which the two substrates can be circularized by the target enzymes the pfTopI dumbbell substrate was added in a 19 fold molar excess compared to the TopI dumbbell substrate in the experiments described in the following. The utilization of this substrate ratio resulted in a number of circularized pfTopI dumbbell substrates sufficient for detection even when in competition with the preferred TopI dumbbell substrate for reaction with pfTopI (see Figure 3).

In order to investigate multiplexed detection of circularized TopI dumbbell substrate and circularized pfTopI dumbbell substrate generated by hTopI and/or pfTopI, both enzymes were added either separately or simultaneously (at stated below) to the reaction tubes containing both substrates as well as the other reaction components for RCA and readout, i.e., Phi29 polymerase, dNTPs and both molecular beacons necessary for detection.

Figure 3A,B show bar charts in which the MV obtained from experiments where either purified hTopI (Figure 3A) or pfTopI (Figure 3B) was added to the sample mixtures described before and the results obtained by measuring fluorescence emission as a function of time using a fluorometer. As evident from Figure 3A and consistent with previous results [8], hTopI reacted with the TopI dumbbell substrate and not on the pfTopI dumbbell substrate, generating increase in CAL-Fluor-Red-590 emission upon RCA. In contrast, pfTopI was able to react with both substrates and incubation with this enzyme resulted in increase of both CAL-Fluor-Red-590 and FAM emission upon RCA. Also in agreement with previous results obtained by the microscopic readout [8] pfTopI tolerated relative high salt concentrations, whereas hTopI was inhibited by 200 mM NaCl (see Figure 3A,B). Since the aim of these investigations was to determine the possible use of the described automated fluorometer readout in future diagnostic setups the main focus was on the detection of pfTopI, which is highly relevant for diagnosis of malaria [9]. The detection limit of pfTopI was determined in titration experiments to approximately 25 nM (significantly different from the negative (*p*: 0.02 from the *t*-test)). Assuming that (1) the *Plasmodium* parasite has a cellular content of pfTopI of approximately 10^5^, which was previously reported for eukaryotic cells [44]; and (2) all enzymes in the preparation were active, the detection limit of 25 nM of pfTopI would correspond to a parasitemia of approximately 500 parasites/μL using a few drops of blood for detection.

It should be noted that the MVs that are plotted in Figure 3A–C were not achieved at the same time (see Figure S2). The MVs for RCA of circularized TopI dumbbell substrates were reached after around 1 h of incubation (T_MV_: ~1 h). For the pfTopI dumbbell substrate on the other hand, the MVs were achieved after around 4 h of incubation (T_MV_: ~4 h). This is interesting since the reaction conditions for RCA of the two dumbbell substrates were identical (happening in the same reaction tube). The reason for this difference in MV remains unresolved but could be due to a difference in the replication speed of the two different circularized substrates by the polymerase due to differences in their secondary structures and sequences. This highlights an important advantage of the real-time detection of RCA compared to the end-point detection necessary when using the microscopic readout. Using end-point measurements the obtained data may not be optimal. Indeed, depending on the chosen time point for end-point analysis, detection of one of the RCA reactions may be suboptimal. By detection of the RCA in real-time based on the development of fluorescence as described here, it is possible to optimize the time window for data collection and ensure that MV has been reached for all reactions under investigation.

### 3.4. Multiplexed Detection of Enzyme Activities from hTopI and pfTopI in the Presence of Complex Human Samples

**Figure 4:**
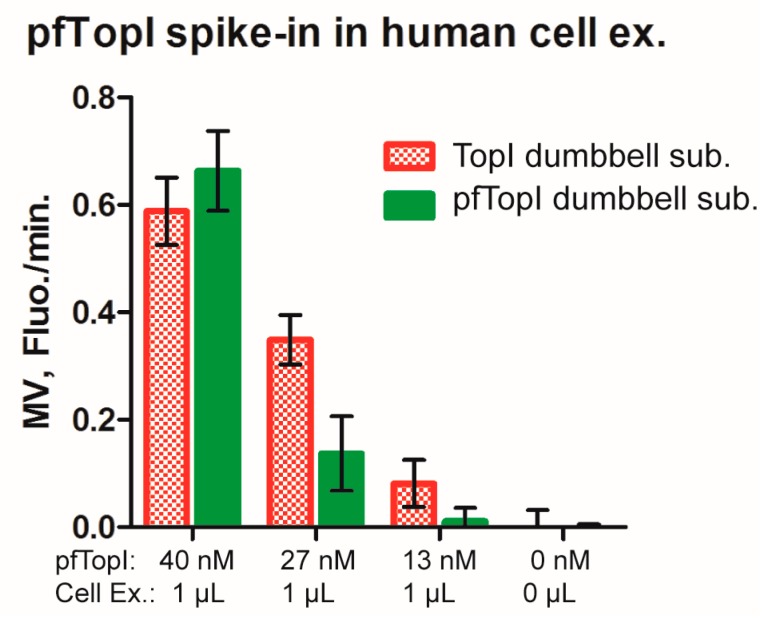
Bar chart showing the mean MV from six experiments where samples including 95 nM pfTopI and 5 nM TopI dumbbell substrates were mixed with cell extract from 50,000 cells and pfTopI as indicated followed by fluorometric analysis. Error bars represent Standard Error of the Mean (SEM) calculated from six experiments. Red bars show CAL-Fluor-Red-590 fluorescence corresponding to RCA of the circularized TopI dumbbell substrate and green bars show FAM fluorescence corresponding to RCA of the circularized pfTopI dumbbell substrate.

To address the potential of the described real-time detection method for malaria diagnostic purposes, different concentrations of the pfTopI enzyme were spiked into reaction mixtures containing nuclear extract from 50,000 cells prepared from human cell lines (used as a mimic of clinical samples from malaria patients) as well as the TopI and pfTopI dumbbell substrates. After incubation to enable circularization of the dumbbell substrates by the enzymes, RCA was performed in the presence of the ID33- and ID16 beacons and analyzed using a fluorometer. The bars in the bar chart shown in Figure 4 represent mean signals that resulted from molecular beacon binding to RCPs generated from circularized TopI dumbbell substrate (red) or pfTopI dumbbell substrate (green). It is evident that the signals from both substrates decreased as a function of decreasing pfTopI concentration. This is consistent with previous reports [8,9] demonstrating that both substrates can be circularized by pfTopI. After addition of pfTopI at a concentration of 12 nM, which is below the detection limit (see Figure 3C), no FAM signal corresponding to unfolding of the ID33 beacon specific to circularization of the pfTopI dumbbell substrate could be observed. In contrast, signal corresponding to ID16 beacon binding to RCPs generated from circularized TopI dumbbell substrates were still significantly above background. This signal can be ascribed RCA of circularized TopI dumbbell substrate present in the utilized cell extracts. The generation of the FAM signal specific to RCA of circularized pfTopI substrate, on the other hand, appeared as a clear indicator of pfTopI in the sample, pointing towards a potential use of the real-time measurement of molecular beacon binding to RCPs in future diagnostic setups.

One interesting thing to note in Figure 4, is the fact that as a unique trait of the sample containing 40 nM of pfTopI and cell extract, the signals resulting from RCA of circularized TopI dumbbell substrate and pfTopI dumbbell substrate are comparable in intensities. The reason for this is unclear but may be the result of binding of competing factors present in the cell extract e.g., DNA repair proteins selectively to the TopI dumbbell substrate, which are characterized by a strand interruption that may recruit repair factors. Such binding would lower the efficient concentration of the TopI dumbbell substrate and could potentially explain the low intensity of signals corresponding to circularization of this substrate relatively to circularized pfTopI dumbbell substrate in the sample with high enzyme concentration. In samples with less added enzyme the efficient concentration of the TopI dumbbell substrate may still be high enough to give the expected result.

## 4. Conclusions

In the present study we demonstrate that two molecular beacons could be used for specific multiplexed detection of two different RCA reactions performed simultaneously in the same reaction tube and that this readout could be supplemented by subsequent microscopic analysis. Furthermore, we showed that the molecular beacon based fluorometric readout method could be used for detection of pfTopI activity in crude biological sample pointing towards a future application in e.g., diagnostic setups.

Moreover, the described setup may add to future development of novel automated systems for fast and high throughput screening of new small molecule drugs targeting pfTopI. With the increasing occurrence of multidrug resistant *Plasmodium* strains fast screening of new small molecule drugs is a priority. Interesting new topoisomerase IB directed drugs targeting other eukaryotic parasites (*Leishmania*) are emerging from surprising new sources [45] and may find use also against malaria.

Measuring of fluorescence over time is a robust method for analyzing RCA reactions. By programming for automatic analyses of the fluorescence data for parameters like MV and T_MV_, it will be relatively easy to analyze multiple samples with minimal data handling. Future development of a model for fitting the primary data might further improve the data acquisition in order to minimize the noise that is often observed at low concentrations of RCA substrates, when using the current method for calculating MV and T_MV_.

## Supplementary Materials

The supplementary materials are available online at http://www.mdpi.com/1424-8220/16/11/1916/s1.
