# Supplementary material for: Optimized Detection of Plasmodium falciparum Topoisomerase I Enzyme Activity in a Complex Biological Sample by the Use of Molecular Beacons
Source: Sensors (Basel). 2016 Nov 15;16(11):1916. doi: 10.3390/s16111916 (PMC5134575; doi:10.3390/s16111916)
Supplement: Supplementary file 1 [file sensors-16-01916-s001.pdf]

Supplementary Materials: Optimized Detection of *Plasmodium falciparum* Topoisomerase I Enzyme Activity in a Complex Biological Sample by the Use of Molecular Beacons

Asger Givskov, Emil L. Kristoffersen, Kamilla Vandsø, Yi-Ping Ho, Magnus Stougaard and Birgitta R. Knudsen

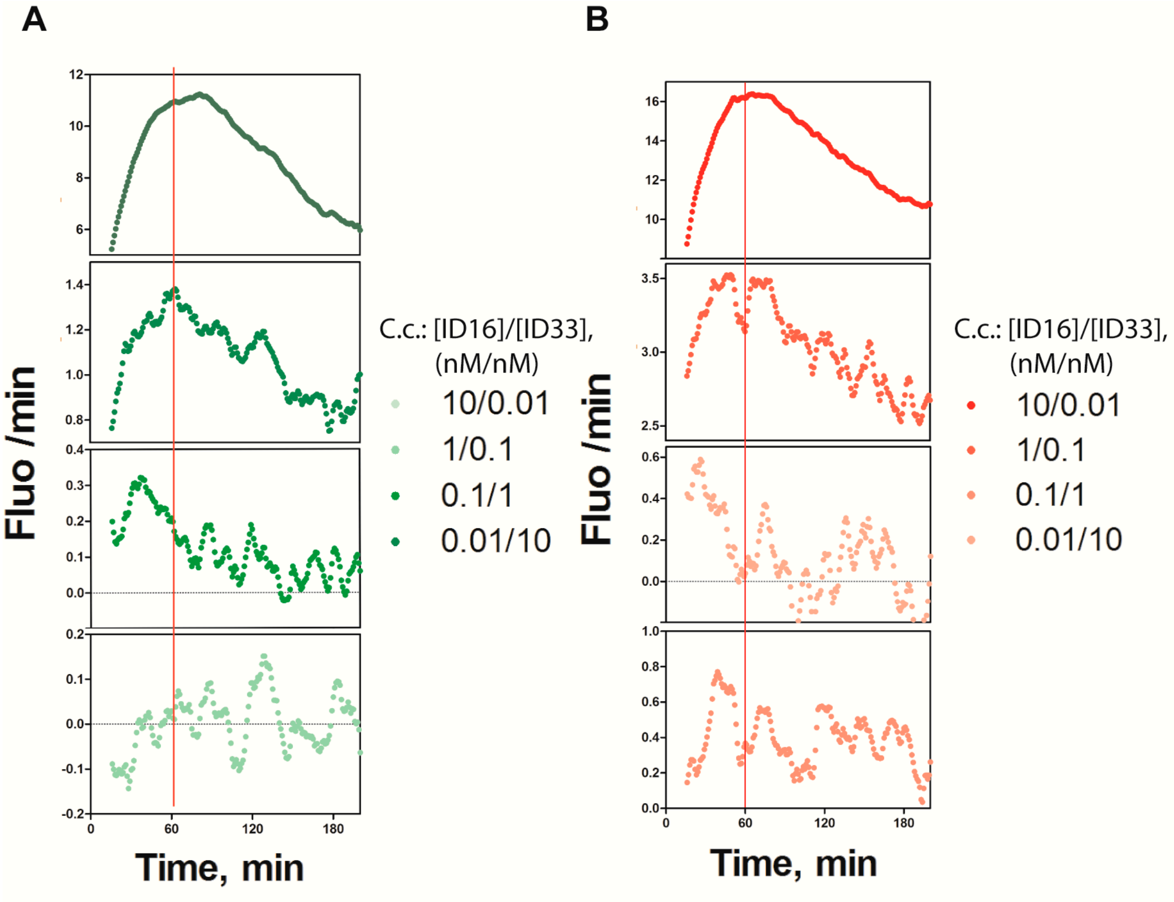

Figure S1. Derived plots from primary data shown in Figure 2A,B of control circle cross titration experiments. These figures are enlargements of the insets in Figure 2A,B.

The T_{MV} Obtained by Real-Time Analysis of RCA of Circles Generated from Different Dumbbell Substrates Differ

During the analysis of all reactions on the dumbbell substrates, it was consistently observed that MV of the RCA was reached at later time points of incubation using the circularized pfTopI dumbbell substrate as template for the reaction compared to using the circularized TopI dumbbell substrate.

In Figure S2, the primary fluorescence data obtained by measuring RCA on the circularized pfTopI dumbbell substrate (Figure S2A) and the TopI dumbbell substrate (Figure S2B), respectively, are shown. (These data were used to calculate MV in Figure 3C for 50 nM of pfTopI enzyme). On the right, derived values calculated from the primary data (as described in the Materials and Methods Section) are plotted as a function of time. From Figure S2A, it is evident that the MV was reached after approximately 4 h ($T_{MV} \approx 4$ h). In Figure S2B, however, the MV was reached after approximately 1 h ($T_{MV} \approx 1$ h), which is about the same time as the MV observed for RCA of the c.c. (shown in Figure 2).

Since the reaction conditions for the two measured reactions were identical (both reactions were performed in the same tube with excess of polymerase), the difference in MV is attributed to the differences between the two dumbbell substrates and their reaction with the polymerase. Note that the T_{MV} were the same for the two molecular beacons, when analyzing the c.c. RCA reactions, (Figure 2), indicating that it is not the molecular beacon binding rate that differs.

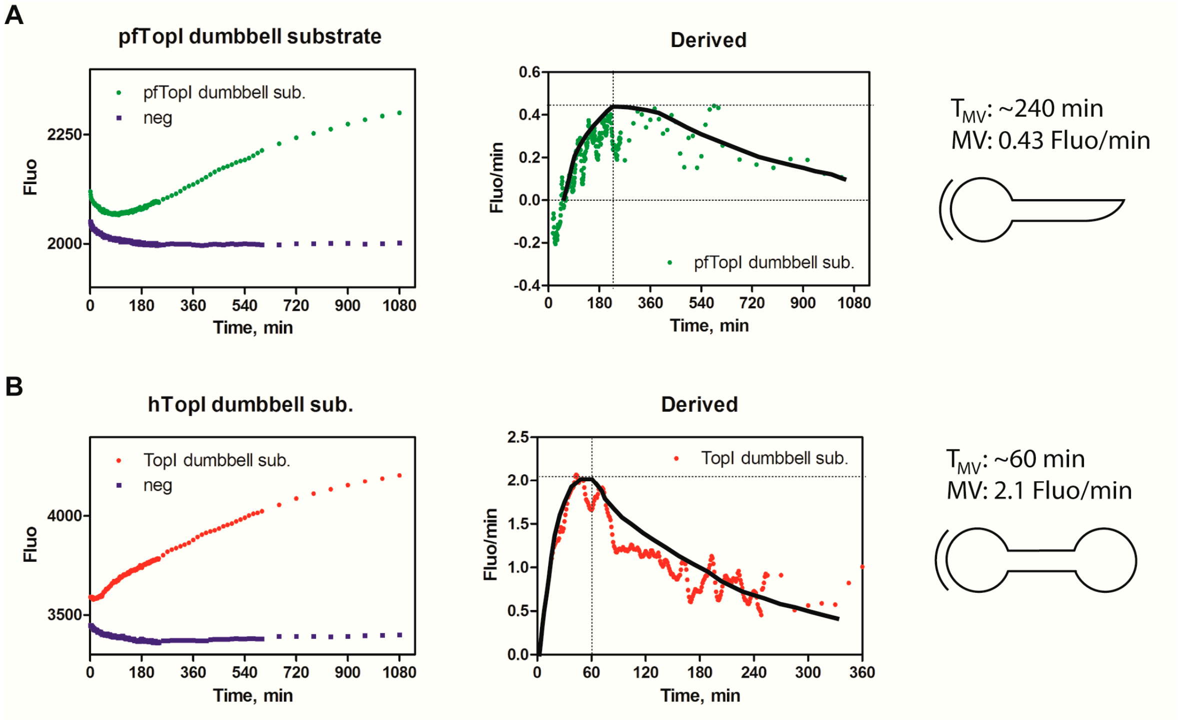

Figure S2. (**A**) Representative FAM fluorescence measurements from experiments where dumbbell substrate mixtures were incubated with 50 nM pfTopI or no pfTopI (the negative control). The graph on the left shows primary results from real-time measurement of RCA over the time span 0 to 1080 min (18 h). The graph on the right shows the derived values calculated for the primary data and plotted as a function of time. The position and time of MV are marked with dashed lines and the values of MV and T_{MV} are noted on the right; (**B**) is similar to (**A**), except that CAL-Flour-Red-590 fluorescence generated by molecular beacon binding to RCPs generated by RCA of circularized TopI substrate was analyzed. The graph on the left shows primary results from RCA incubation for 0 to 360 min (6 h). The graph on the right shows the TAMRA fluorescence-derived values plotted as described in A. The black line in the plots indicates the trend of the data. To the right of the plots, schematic drawings portray the dumbbell substrates (pfTopI dumbbell substrate above, TopI dumbbell substrate below), as well as the calculated MV and T_{MF} values for the given data set.
